# Hospital Influenza Outbreak Management in the Post-COVID Era: A Narrative Review of Evolving Practices and Feasibility Considerations

**DOI:** 10.3390/healthcare14010050

**Published:** 2025-12-24

**Authors:** Wei-Hsuan Huang, Yi-Fang Ho, Jheng-Yi Yeh, Po-Yu Liu, Po-Hsiu Huang

**Affiliations:** 1 Division of Infectious Diseases, Department of Internal Medicine, Taichung Veterans General Hospital, Taichung 407, Taiwan; hpluffy@gmail.com (W.-H.H.); liupoyu@gmail.com (P.-Y.L.); 2 Infection Control Center, Taichung Veterans General Hospital, Taichung 407, Taiwan; yfh02@vghtc.gov.tw (Y.-F.H.); jen773@vghtc.gov.tw (J.-Y.Y.); 3 School of Medicine, National Yang Ming Chiao Tung University, Taipei 112, Taiwan; 4 Department of Post-Baccalaureate Medicine, College of Medicine, National Chung Hsing University, Taichung 402, Taiwan

**Keywords:** hospital-acquired influenza, nosocomial transmission, outbreak management, nucleic acid amplification testing, antiviral therapy and chemoprophylaxis, healthcare worker vaccination

## Abstract

**Highlights:**

**What are the main findings?**

- Hospital influenza outbreak control has shifted significantly post-COVID, with layered measures (N95 use, ventilation, multiplex PCR) now standard, reducing nosocomial transmission.
- Quantitative figures and tables (predictive values of diagnostic tests, outbreak thresholds, and intervention schematics) provide evidence-based visuals for clinicians and infection-control teams

**What are the implication of the main findings?**

- Visual, data-driven tools enable faster recognition and standardized response to influenza outbreaks, helping frontline teams translate guidelines into action.
- Hospitals can adapt COVID-era practices into routine seasonal influenza playbooks, preserving workforce capacity and protecting vulnerable patients.

**Abstract:**

**Background**: Hospital-acquired influenza remains a persistent threat that amplifies morbidity, mortality, length of stay, and operational strain, particularly among older and immunocompromised inpatients. The COVID-19 era reshaped control norms—normalizing N95 use during surges, ventilation improvements, and routine multiplex PCR—creating an opportunity to strengthen hospital outbreak management. **Methods**: We conducted a targeted narrative review of WHO/CDC/Infectious Diseases Society of America (IDSA) guidance and peer-reviewed studies (January 2015–August 2025), emphasizing adult inpatient care. This narrative review synthesizes recent evidence and discusses theoretical implications for practice, rather than establishing formal guidelines. Evidence was synthesized into pragmatic practice statements on detection, diagnostics, isolation/cohorting, antivirals, chemoprophylaxis, vaccination, surveillance, and communication. **Results**: Early recognition and test-based confirmation are pivotal. For inpatients, nucleic-acid amplification tests are preferred; negative antigen tests warrant PCR confirmation, and lower-respiratory specimens improve yield in severe disease. A practical outbreak threshold is ≥2 epidemiologically linked, laboratory-confirmed cases within 72 h on the same ward. Effective control may require immediate isolation or cohorting with dedicated staff, strict droplet/respiratory protection, and daily active surveillance. Early oseltamivir (≤48 h from onset or on admission) reduces mortality and length of stay; short-course post-exposure prophylaxis for exposed patients or staff lowers secondary attack rates. Integrated vaccination efforts for healthcare personnel and high-risk patients reinforce workforce resilience and reduce transmission. **Conclusions**: A standardized, clinician-led bundle—early molecular testing, do-not-delay antivirals, decisive cohorting and Personal protective equipment (PPE), targeted chemoprophylaxis, vaccination, and disciplined communication— could help curb transmission, protect vulnerable patients and staff, and preserve capacity. Hospitals should codify COVID-era layered controls for seasonal influenza and rehearse unit-level outbreak playbooks to accelerate response and recovery. These recommendations target clinicians and infection-prevention leaders in acute-care hospitals.

## 1. Introduction

Nosocomial influenza remains a significant threat within healthcare settings due to rapid transmission and severe clinical outcomes, particularly affecting vulnerable patient populations. Hospital influenza outbreaks contribute substantially to patient morbidity, mortality, prolonged hospitalization, and increased healthcare costs. Globally, influenza causes approximately 3–5 million cases of severe illness and 290,000–650,000 deaths each year [1,2]. Hospitalized patients face heightened risks due to susceptibility and complications such as pneumonia and exacerbation of chronic illnesses. In the United States alone, the CDC estimates 9–41 million influenza illnesses per year [1,2,3]. Global estimates are provided to contextualize the worldwide burden of influenza, whilst U.S. data, derived from a well-established, hospital-based surveillance system, exemplify the operational magnitude and healthcare impact pertinent to acute-care environments. Thus, rapid identification and prompt intervention are crucial to mitigating influenza outbreaks in hospitals.

Clinicians play a central role in early detection and response to hospital-based influenza outbreaks. Key responsibilities include maintaining a high index of suspicion for influenza, promptly ordering diagnostic testing, and initiating isolation precautions for suspected cases. Additionally, clinicians are encouraged to commence antiviral therapy, actively participate in infection control measures, and collaborate with multidisciplinary teams to manage outbreaks effectively [4,5,6]. Timely clinical action can significantly limit transmission, protect healthcare personnel, and reduce the severity and duration of outbreaks.

Notably, the management of hospital influenza outbreaks fundamentally constitutes a multidisciplinary and organizational endeavor rather than a solitary clinical decision. Successful control relies on meticulous coordination among frontline clinicians, infection prevention and control (IPC) teams, clinical microbiology laboratories, nursing leadership, hospital administration, and occupational health services. While clinicians frequently identify index cases and initiate diagnostic testing, IPC teams are responsible for outbreak declaration, surveillance, cohorting decisions, and implementing control measures at the unit level. Microbiology laboratories serve a pivotal function by ensuring the prompt processing of molecular diagnostics and effectively communicating results that directly initiate operational measures. Hospital leadership, in turn, supports resource allocation, staffing modifications, and policy enforcement. Without this coordinated, systemic response, even timely clinical recognition may not suffice to prevent prolonged nosocomial transmission.

This review synthesizes current evidence and guideline recommendations on early detection, diagnostics, and outbreak management in hospital settings. Although burnout and outbreak response principles have been discussed across various professional and institutional contexts, hospital settings involve distinct clinical, ethical, and system-level constraints, particularly patient safety responsibilities, infection exposure, and workforce dependency, that necessitate healthcare-specific evidence. The discussion will specifically focus on strategies for early case identification, diagnostic methodologies, isolation and cohorting practices, appropriate use of antiviral medications, and coordinated outbreak control measures. In addition, we contrast pre-COVID practices, including droplet precautions and limited point-of-care tests, with post-COVID norms, including N95 source control during surges, routine multiplex PCR, and multidisciplinary surge plans.

A narrative review was chosen to integrate heterogeneous post-COVID influenza practices and variable evidence on early detection and hospital outbreak management, synthesizing existing guidance rather than proposing new recommendations.

Accordingly, the objective of this narrative review is to synthesize post-2015 evidence and major international guidelines to clarify how hospital influenza outbreaks are detected, operationally defined, and managed in the post-COVID era. Specifically, we aim to (1) summarize practical approaches to early detection and diagnostic testing in hospitalized patients, (2) examine commonly used outbreak thresholds and infection-control interventions, and (3) critically appraise how guideline recommendations translate into real-world hospital practice across different institutional contexts.

## 2. Methods

We conducted a targeted narrative review focused on influenza management in acute-care hospital settings. Primary sources included WHO, CDC, and IDSA guidance documents, as well as peer-reviewed literature indexed in PubMed and Embase from January 2015 to August 2025. Additional materials were identified through WHO IRIS, CDC Stacks, and manual searching of reference lists from key articles and guidelines.

### 2.1. Search Strategy

A structured Boolean search was applied and adapted for each database using combinations of the following terms: (influenza OR “influenza A”) AND (hospital OR nosocomial OR “healthcare-associated”) AND (outbreak OR transmission OR cohorting OR isolation) AND (PCR OR NAAT OR diagnosis OR oseltamivir OR “post-exposure prophylaxis”).

Search strategies were refined iteratively to capture literature on outbreak thresholds, rapid diagnostics, cohorting practices, antiviral treatment, chemoprophylaxis, and vaccination programs relevant to inpatient settings. These domains were selected a priori because they represent the most consistently reported and clinically actionable outcomes in hospital influenza outbreak management.

### 2.2. Eligibility Criteria

Studies were included if they met all of the following inclusion criteria:

- Examined hospital-acquired or healthcare-associated influenza;
- Evaluated outbreak management, transmission-control strategies, diagnostics, antiviral therapy, chemoprophylaxis, or inpatient vaccination;
- Involved adults or mixed adult–pediatric inpatient settings;
- Were published in English between 2015 and 2025;
- Represented guideline statements, randomized trials, cohort studies, outbreak investigations, or systematic reviews.

Studies were excluded if they met any of the following exclusion criteria:

- Pediatric-only community studies;
- Non-English publications;
- Abstracts, letters, or opinion pieces without primary data;
- Laboratory virology studies without direct clinical relevance.

### 2.3. Screening and Study Selection

Two authors independently screened titles and abstracts, followed by full-text review. Disagreements were resolved by consensus. In total, 487 records were identified, 162 full texts were assessed, and 87 articles met the inclusion criteria and were retained for synthesis.

**Figure 1:**
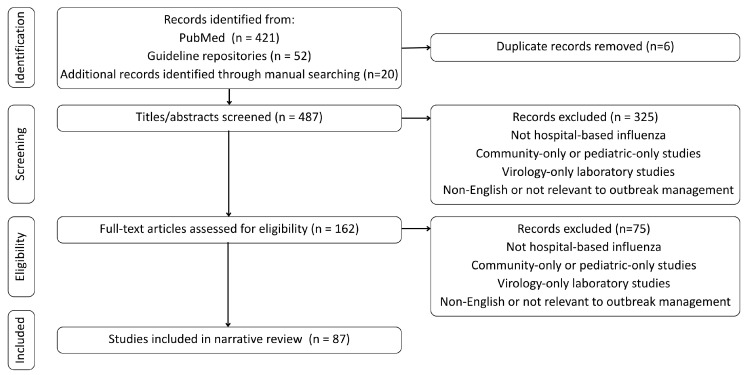
Study identification and selection flow diagram for the targeted narrative review.

A PRISMA-style flow diagram of the identification, screening, eligibility, and inclusion process is shown in Figure 1. Although a PRISMA-style flow diagram was used to enhance transparency in study identification and selection, this review was intentionally conducted as a narrative review to synthesize heterogeneous guideline documents, outbreak investigations, and observational studies, rather than as a formal systematic review requiring standardized risk-of-bias assessment or quantitative synthesis.

### 2.4. Data Extraction and Synthesis

From each included study, we extracted key methodological and clinical variables, including the study design, setting, population characteristics, diagnostic approach, and the type of intervention evaluated (e.g., cohorting strategies, PPE practices, or antiviral prophylaxis). Relevant outcomes, such as transmission rates, secondary attack rates, mortality, and other measures of effectiveness, were also collected. Evidence from adult inpatient settings was prioritized, and data from long-term care facilities were considered only when they provided meaningful analogs to hospital-based outbreak management. Findings were synthesized thematically and organized into 12 practice statements, with the strength of each statement classified pragmatically as high, moderate, or conditional based on guideline convergence and the overall quality and consistency of the supporting evidence.

### 2.5. Quality Appraisal and Limitations

As a narrative review, this study has inherent limitations. The synthesis relies on heterogeneous study designs and guideline documents, precluding formal meta-analysis and increasing the risk of publication bias. Evidence appraisal was pragmatic rather than systematic, and the strength of practice statements reflects guideline convergence and consistency rather than formal grading. Formal funnel plot analysis was not performed, as the included literature was not amenable to quantitative synthesis; however, the evidence base was largely dominated by observational studies and outbreak reports describing successful or actionable interventions, suggesting a potential publication bias toward positive findings.

Several limitations related to the underlying data sources should be noted. International and national guidelines (e.g., WHO, CDC, IDSA) are largely consensus-based and may incorporate expert opinion in areas where high-quality comparative evidence is limited. Surveillance and outbreak reports often reflect local testing practices, reporting thresholds, and resource availability rather than true incidence or transmission dynamics. In addition, many hospital outbreak studies are single-center and retrospective, and are often conducted in high-resource tertiary settings, which may limit external validity.

Substantial heterogeneity existed across included studies in terms of study design (randomized trials, cohort studies, outbreak investigations), patient populations (general wards, intensive care units, immunocompromised hosts), diagnostic approaches, and outcome measures (e.g., secondary attack rates, length of stay, mortality, or healthcare worker absenteeism).

In addition, data from low- and middle-income countries remain limited, potentially limiting generalizability across healthcare settings.

Accordingly, the findings of this review should be interpreted as a synthesis of convergent themes and feasibility considerations rather than prescriptive or universally applicable standards. These limitations are acknowledged when interpreting the findings.

## 3. Results

A total of 87 studies met the inclusion criteria, including 7 randomized/quasi-experimental trials, 38 cohort studies, 12 outbreak investigations, 14 diagnostic accuracy studies, 6 systematic reviews, and 10 major guideline documents—most involved adult inpatients in acute-care hospitals across North America, Europe, and Asia. Based on convergent findings across the included literature, these studies addressed early detection, diagnostics, isolation/cohorting, PPE, antiviral therapy, and chemoprophylaxis, consistently supporting rapid molecular testing, early antivirals, timely isolation, and structured outbreak responses.

While multiple post-COVID practices are associated with reductions in healthcare-associated respiratory viral infections, much of the supporting evidence is derived from descriptive institutional reports, outbreak investigations, or before–after observations rather than controlled comparative studies. Consequently, observed effectiveness and variability should be interpreted as context-dependent and hypothesis-generating rather than definitive causal estimates.

### 3.1. The Post-COVID Paradigm Shift in Respiratory Virus Control

#### 3.1.1. Transmission Precautions

Before 2020, hospital influenza control primarily relied on established droplet-based precautions and seasonal surveillance strategies aligned with predictable winter outbreaks. Building on these traditional norms, the COVID-19 pandemic prompted a shift toward layered respiratory protection, including expanded use of N95 masking during respiratory surges and investments in ventilation improvements. As described above, these COVID-era institutional practices have since been extended to influenza control and, in several institutional reports, have been associated with reduced nosocomial transmission [7].

Emerging epidemiologic studies suggest measurable post-COVID shifts in influenza transmissibility. During 2020–2021, universal masking, reduced mobility, and enhanced ventilation nearly eliminated seasonal influenza circulation globally [8], and many hospitals reported zero hospital-acquired influenza cases. As masking mandates relaxed, influenza re-emerged, yet several metrics indicate altered transmission dynamics compared with pre-COVID periods.

Household generation-time studies in the United States found slightly longer mean generation times (≈3.9–4.2 days vs. historical 2.6–3.2 days) and lower secondary attack rates during periods of high mask adherence [9]. Healthcare data similarly show reduced healthcare-worker attack rates (<2%) in settings that maintain universal masking or N95 use, compared with historical pre-COVID outbreaks, which reported attack rates of 11–59% [10]. Multiple institutions have also documented fewer and shorter influenza outbreaks after 2020, with rapid Nucleic Acid Amplification Tests (NAAT)-based detection and early cohorting frequently suppressing in-facility Rt below 1 under layered protections [11,12].

Taken together, these quantitative observations suggest that COVID-era behavioral and environmental modifications—improved ventilation, reduced presenteeism, heightened PPE adherence, and early molecular testing—have reshaped the operational and epidemiologic profile of seasonal influenza in acute-care settings.

#### 3.1.2. Clinical Recognition and Diagnosis

Clinicians once relied on classic symptom patterns, including abrupt onset of fever, cough, and myalgia, as sufficient for diagnosing influenza during the peak season. Since COVID-19 blurred the clinical boundaries between respiratory viruses, reliance on classic symptom patterns has become less reliable. Coinfections (e.g., influenza with SARS-CoV-2 or RSV) and overlapping presentations have been increasingly recognized, making syndromic diagnosis challenging [13]. Consequently, many clinicians and hospitals now emphasize or have shifted toward laboratory confirmation using rapid molecular assays or multiplex PCR, particularly in high-risk inpatients [14]. While the virus’s incubation and infectivity profiles have remained biologically stable, the diagnostic threshold has shifted toward test-based confirmation, rather than relying solely on clinical judgment.

#### 3.1.3. Infection Control Policies

Hospital infection-control protocols for influenza were traditionally moderate; symptomatic staff were furloughed briefly, isolation precautions often ended once fever subsided, and universal PPE use was uncommon. The COVID-19 experience prompted many institutions to adopt more stringent isolation policies, with some applying SARS-CoV-2-like criteria, such as isolation until full symptom resolution or for at least seven days, for patients with confirmed influenza [3]. However, implementation varies across facilities depending on infrastructure and staffing resources. Hospitals also intensified attention to pre-symptomatic transmission and adopted conservative staff sickness policies. Practices such as routine staff cohorting, active daily surveillance, and extended masking for healthcare workers, introduced during COVID, have persisted and are now applied to seasonal influenza response [5].

Based on guideline comparisons and institutional reports, the 2020 norms emphasized droplet/contact precautions with airborne measures for Aerosol-Generating Procedures (AGPs) only. COVID-19 established the utility of layered controls (N95s during respiratory surges, ventilation upgrades, universal masking), which are now commonly extended to influenza and associated with reduced nosocomial spread [7].

Based on published diagnostic and outbreak reports, syndromic diagnosis is less reliable due to symptom overlaps and co-infections (e.g., SARS-CoV-2, RSV) [13]. There is greater reliance on rapid molecular assays/multiplex PCR, especially in high-risk populations [14].

Across guidelines and institutional policies reviewed, isolation often continues until full symptom resolution or ≥7 days, with stricter staff illness policies, routine cohorting, and active daily surveillance—COVID-era rigor now codified for seasonal influenza [3,5]. Table 1 contrasts pre- and post-COVID-19 influenza management in hospitals, illustrating how pandemic-era lessons have reshaped transmission precautions, clinical recognition, and outbreak containment. These shifts reflect not only improved understanding of air-borne dynamics but also institutional responses that now prioritize layered controls and enhance diagnostic sensitivity.

**Figure 2:**
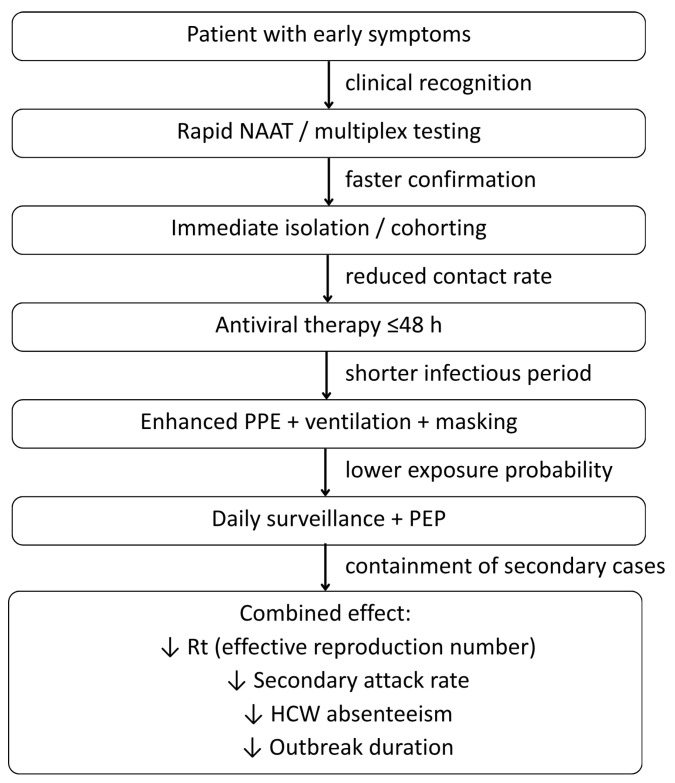
Conceptual framework linking early detection, diagnostics, and layered infection-control interventions to reduction in hospital influenza transmission. Directional arrows denote the hypothesized causal sequence and intermediate effects leading to reduced transmission and improved outbreak control.

In our synthesis, we propose a conceptual framework (Figure 2) that illustrates how early clinical recognition, rapid molecular diagnostics, and layered infection-control measures interact to influence epidemiological outcomes in hospital settings. In this model, timely NAAT-based diagnosis shortens the interval from symptom onset to isolation, while antiviral therapy reduces the duration of infectiousness. Additionally, interventions such as N95 use, ventilation improvements, and staff cohorting lower the probability of exposure. These combined effects act on core transmission parameters, reducing the effective reproduction number (Rt), secondary attack rates, and healthcare worker absenteeism, thereby compressing outbreak duration and preventing escalation. This integrative framework provides a unifying structure that links the post-COVID paradigm shift to measurable transmission outcomes, setting the foundation for the operational strategies discussed in the next section.

While these observations reflect post-pandemic trends reported in higher-resource hospital settings, implementation remains heterogeneous globally. Adoption depends on local infrastructure, workforce capacity, and the maturity of infection control.

Early symptom recognition prompts rapid NAAT or multiplex testing, enabling faster laboratory confirmation and immediate isolation or cohorting to reduce contact opportunities. Early antiviral therapy shortens the infectious period, while enhanced PPE, ventilation improvements, and masking lower exposure probability. Daily surveillance and post-exposure prophylaxis (PEP) help contain secondary cases. Collectively, these mechanisms reduce the effective reproduction number (Rt), ward-level secondary attack rates, healthcare-worker absenteeism (HCW), and overall outbreak duration.

### 3.2. Outbreak Detection and Surveillance

#### 3.2.1. Early Recognition and Reporting Matter

Hospitalized patients, especially older adults, immunocompromised persons, and those with major comorbidities, are highly vulnerable to influenza complications. Ward outbreaks carry substantial mortality, with pooled hospital-acquired influenza mortality around 16% based on recent multicenter analyses [15,16], and reported case-fatality rates ranging from 14 to 71% among ICU or transplant-unit patients. Early recognition enables immediate control measures, including single-room isolation or cohorting, meticulous hand hygiene and PPE, and antiviral chemoprophylaxis for exposed contacts [5]. CDC recommends offering antiviral prophylaxis to all exposed residents during long-term care outbreaks; in our synthesis, a comparable approach may be appropriate for high-risk hospital units [5]. These steps reduce incident cases and deaths and prompt rapid testing and treatment of other symptomatic patients on the unit.

Unchecked outbreaks rapidly erode staffing. In prior hospital outbreaks, healthcare-worker (HCW) attack rates reached 11–59% when precautions were inadequate [10]. High vaccination coverage, exclusion of symptomatic staff, and strict adherence to PPE can reduce HCW attack rates to below 2%, as reported in hospitals implementing stringent droplet/respiratory precautions during influenza seasons [10]. Early recognition and staff notification could enable prompt furlough of symptomatic exposed personnel, timely prophylaxis or early therapy, and renewed emphasis on PPE—potentially helping to prevent cascading absenteeism and averting admission holds or patient transfers driven by workforce shortages. Swift control preserves operational capacity; delays can turn a small cluster into a staffing crisis.

Timely reporting ensures legal compliance and unlocks support from public health authorities. Early notification facilitates epidemiologic assistance (e.g., genotyping, source investigation), response coordination (e.g., emergency antiviral stockpiling, staffing support), and transparent communication with patients and their families. Proactive reporting signals a commitment to safety; missed or late recognition undermines trust and increases liability risk.

Define an outbreak with two linked cases and take immediate action. Clinically, rapid control reduces preventable harm in high-risk inpatients; operationally, it preserves staffing and core services—a lesson reinforced by the COVID-19 era.

#### 3.2.2. Early Detection and Surveillance in the Hospital Setting

During influenza season, suspect influenza in any hospitalized patient with new respiratory or systemic symptoms—particularly the elderly and immunocompromised—even when classic features are absent [17,18,19]. As discussed above, atypical or afebrile presentations are common in these populations; therefore, early clinical suspicion should prompt immediate testing and isolation rather than anchoring on alternative diagnoses.

Upon initial suspicion (e.g., new cough/malaise, or unexplained confusion with low-grade fever in an older adult), initiate droplet precautions immediately and isolate the patient [5]. Notify IPC without delay to enable unit-level surveillance, cohorting, admission restrictions, and other measures. Small clusters—commonly defined as ≥2 epidemiologically linked, laboratory-confirmed cases within 72 h on the same ward—can reasonably be considered potential outbreaks. This operational threshold is adapted from the CDC’s long-term care facility (LTCF) outbreak guidance and is widely applied in hospital infection-prevention protocols, prompting the initiation of outbreak response measures when feasible [5]. Order influenza testing once during flu season; rapid confirmation supports timely antivirals, isolation, and outbreak recognition. Vigilant bedside detection of the index case and subsequent cases—paired with rapid isolation and IPC notification—can markedly limit the spread [3].

In pediatric units, outbreak detection and control require additional adaptation beyond standard adult-focused protocols. Close-contact care, routine caregiver presence, and higher viral shedding complicate isolation and cohorting decisions. Practical adaptations include cohorting infected children together with their designated caregivers, emphasizing caregiver masking and education, and applying lower thresholds for testing symptomatic children and exposed caregivers during suspected outbreaks.

#### 3.2.3. Diagnostic Testing for Influenza in Hospitals

Role of testing

Rapid, accurate diagnosis enables timely antivirals, appropriate isolation, and antimicrobial stewardship. As discussed above, because syndromic diagnosis is unreliable in hospitalized patients, current guidelines recommend nucleic acid amplification tests (NAATs) as first-line assays for inpatients [4].

Antigen tests vs. NAATs

Rapid influenza diagnostic tests (RIDTs) yield results in ~15 min but have low–moderate sensitivity (~50–70%) despite high specificity (~95–99%) [20]. Although FDA standards now require ≥80% sensitivity and newer assays perform better [21], NAATs (e.g., RT-PCR) are preferred for all hospitalized patients. If an antigen test is used and negative, confirm with PCR [4].

**Figure 3:**
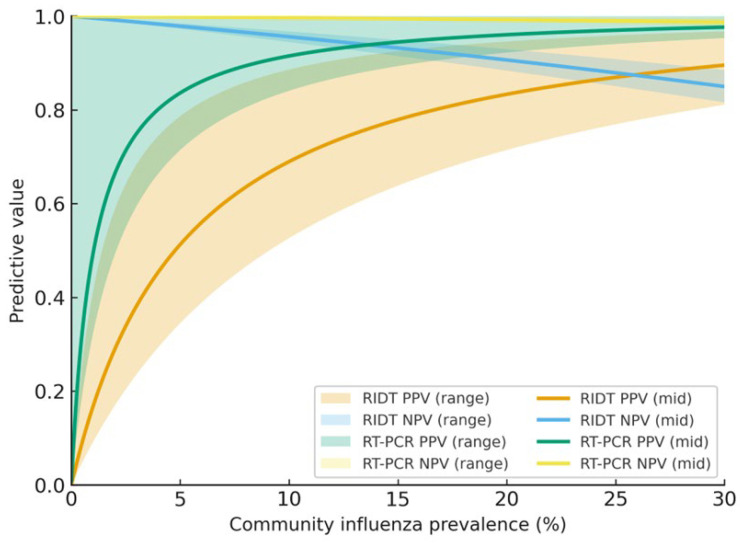
Predictive values vs. prevalence (literature-based ranges).

Rapid molecular assays. Near-patient NAATs (≈15–30 min) approach laboratory RT-PCR in accuracy and shorten time to diagnosis, reduce unnecessary antibiotics, and improve antiviral stewardship; many hospitals deploy them at triage or in EDs [4]. As shown in Figure 3, predictive values for RIDTs and RT-PCR change substantially with community prevalence: PPV drops in low-prevalence settings despite high specificity, whereas NPV declines at peak prevalence due to imperfect sensitivity.

Operational factors, including platform cost, test availability, staffing, and laboratory turnaround times, also shape real-world testing policies. Many hospitals balance the use of rapid NAATs for time-critical decisions with centralized PCR to reduce overall costs, leading to hybrid algorithms that optimize both accuracy and feasibility.

Positive predictive values (PPV) and negative predictive values (NPV) for RIDTs and RT-PCR are plotted against community influenza prevalence (0–30%). Shaded bands reflect plausible PPV/NPV intervals derived from pooled estimates and guidance: RIDT (antigen) sensitivity 0.50–0.70 and specificity 0.95–0.99; RT-PCR sensitivity 0.95–0.99 and specificity 0.98–1.00 [20,21,22,23,24]. Median reference curves are overlaid for readability. At low prevalence, PPV decreases despite high specificity (risk of false positives); at peak season, higher prevalence lowers NPV because of imperfect sensitivity (risk of false negatives). Unexpected or discordant results should be confirmed with molecular testing, as recommended by the CDC/ADLM [21,22,23]. Abbreviations: RIDT, rapid influenza diagnostic test; RT-PCR, reverse transcriptase polymerase chain reaction; PPV, positive predictive value; NPV, negative predictive value.

Multiplex panels

Panels that detect influenza A/B, SARS-CoV-2, RSV, and other viruses from a single swab are endorsed for immunocompromised inpatients (A-III) and may aid broader cohorting and de-escalation decisions in others. Concurrent testing for influenza and SARS-CoV-2 is encouraged when feasible [3,4,6].

Specimen collection

Obtain a nasopharyngeal (or combined nasal/throat) swab as early as possible—ideally within 3–4 days of symptom onset [4]. In patients with pneumonia or on mechanical ventilation, test lower respiratory tract specimens (endotracheal aspirate or BAL), as upper-tract swabs may be falsely negative; severely ill or immunocompromised patients may shed longer and at higher titers in the lower airways. Repeat lower-tract PCR when clinical suspicion remains high despite an initial negative upper-tract test [4,25].

In patients with chronic diseases or immunocompromising conditions, outbreak management often requires tailored diagnostic and isolation strategies. Prolonged viral shedding and atypical clinical presentations may necessitate earlier molecular testing, repeat NAATs, and, when feasible, preferential testing of lower respiratory tract specimens. In outbreak settings, extended isolation durations and cautious de-escalation of transmission-based precautions may be warranted despite clinical improvement, particularly in oncology, transplant, and dialysis units where the consequences of onward transmission are substantial.

Serology and Viral Culture (Non-Recommended Methods)

Importantly, serologic testing for influenza has no role in acute diagnostics and is not recommended (it is unreliable and too slow for guiding care) [4]. Viral culture is used only for research or public health surveillance purposes (e.g., strain typing) and is not practical for routine diagnosis due to the prolonged time to results [22].

Clinical interpretation

During flu season, test inpatients with compatible illnesses, particularly those at high risk, using NAATs. A positive result supports immediate antivirals and continued precautions; a high-quality negative may permit de-escalation depending on context. With very high pretest probability or an active outbreak, consider repeat testing and maintain precautions. Do not delay empiric antivirals in critically ill patients while awaiting results [4]. Effective testing, coupled with prompt isolation and treatment, improves outcomes and limits in-hospital transmission [4,6,16].

#### 3.2.4. Interpreting Test Results in Clinical Context

Contextual Interpretation

Interpreting influenza assays in hospitals requires coupling laboratory results with clinical probability and epidemiology; predictive value varies with community activity, symptom profile, specimen timing, and source [23].

Positive Results

During peak seasons or institutional clusters, positive antigen or molecular test results are highly likely to be accurate [21]. Given the high specificity of rapid antigen tests (≈95–99%), false positives are uncommon when prevalence is high. In low-activity periods, confirm unexpected positives with RT-PCR. Shortly after intranasal live-attenuated vaccination, a positive result may reflect vaccine virus rather than wild-type infection [21].

Negative Results

A single negative test, particularly a rapid antigen test, does not exclude influenza when pretest probability is moderate to high. RIDTs detect only a subset of actual cases, so false negatives are frequent in-season; initiate antivirals when clinical suspicion is high and obtain a confirmatory molecular assay rather than withholding treatment solely based on a negative RIDT [21,24].

Preferred Modality (RT-PCR/NAATs)

RT-PCR (or other NAATs) is preferred for all hospitalized patients with suspected influenza owing to superior sensitivity and specificity; multiplex panels are recommended for immunocompromised inpatients to assess co-infection [4,21]. Rapid cartridge-based NAATs provide PCR-level accuracy within 15–30 min and support time-critical decisions [26,27].

Timing of Collection

The diagnostic yield is highest within 3–4 days of symptom onset; later sampling increases the risk of false negatives, particularly for antigen assays. PCR remains positive longer but ultimately turns negative as the infection resolves. Collect specimens as early as possible. For late presenters with a compatible illness and a negative test, integrate clinical and epidemiologic context when determining treatment and isolation [21,23,28].

Specimen Type and Site

Nasopharyngeal (or combined nasal/throat) swabs are standard in the absence of pneumonia, whereas lower-respiratory specimens (endotracheal aspirate or BAL) should be obtained in severe lower-tract disease or when upper-tract testing is negative despite high suspicion; some infections are detectable only in the lower airways. Use assays validated for the sampled site and ensure proper technique/transport to optimize accuracy [21,23,27,29,30].

Immunocompromised Hosts

Symptoms may be attenuated, and viral shedding can persist for weeks to months; PCR positivity may represent ongoing infection or residual RNA and must be interpreted clinically. Maintain a low threshold for repeat or alternative-site testing, favor multiplex NAATs, and start antivirals when suspicion is high, even if initial rapid testing is negative [4,21,31,32].

Outbreak Settings

In suspected ward outbreaks, act immediately on positives (cohorting, PPE reinforcement, antivirals) and confirm negative screens with NAATs to avoid missed cases. Test multiple symptomatic patients and interpret negative RIDTs cautiously until molecular results are available [4,21].

### 3.3. Infection Control Measures

#### 3.3.1. Adherence to Institutional Algorithms and Protocols

Clinician adherence to hospital influenza testing algorithms and outbreak protocols is likely essential to effective control. Early in the season, once community transmission is established, institutions typically recommend testing patients with compatible symptoms, such as fever with cough or any acute respiratory illness, to expedite identification, isolation, and treatment in line with local policy [32]. This protocol fidelity anchors downstream actions across diagnostics, placement, prophylaxis, and communication, reducing diagnostic delay and preventing silent transmission within wards.

#### 3.3.2. Expanded Testing in Confirmed Outbreaks

During a confirmed hospital outbreak, testing thresholds are appropriately lowered and surveillance is intensified to capture every potential case. Daily active surveillance of patients—and, where specified, staff—on affected units aims to detect ILI, fever, or atypical manifestations (e.g., delirium in older adults) at the earliest opportunity [3,5]. Case definitions broaden in this phase; for example, some guidelines recommend testing for any patient with one or more acute respiratory symptoms, isolated fever, or altered mental status, reflecting the higher pretest probability and atypical presentations in vulnerable populations [4,5].

#### 3.3.3. Diagnostic Strategy and Follow-Up Testing

Outbreak algorithms should prioritize molecular assays (NAAT/RT-PCR) over rapid antigen tests due to superior sensitivity in detecting early and mild cases. When an initial test is negative but clinical suspicion remains, protocols commonly advise a confirmatory RT-PCR or repeat sampling within 24–48 h to mitigate false-negative results [5]. Rapid confirmation supports timely cohorting and targeted antiviral treatment. Protocols frequently include chemoprophylaxis for exposed patients and staff once nosocomial transmission is recognized [4]. Diligent execution by clinicians—ordering repeat tests or additional specimens when indicated—ensures missed cases are minimized.

#### 3.3.4. Outbreak Recognition and Response Activation

Many institutions define an influenza outbreak as ≥2 epidemiologically linked, laboratory-confirmed cases on the same ward within 72 h, or a sudden rise in ILI above the unit’s baseline [5]. Meeting this threshold triggers IPC to activate incident command, alert frontline teams, and initiate post-exposure chemoprophylaxis as appropriate actions that compress the window for onward transmission and standardize decision-making under pressure [5].

#### 3.3.5. Patient Placement, Isolation, and Cohorting

As soon as an outbreak is declared, infected patients should be moved to single rooms; when capacity is limited, cohorted bays may be used, provided all patients share the same influenza strain and dedicated staff are assigned [3,32]. A prospective Swiss study of a “Droplet Precautions on-site” (DroPS) model reported comparable nosocomial influenza rates to those of traditional single-room isolation, while preserving scarce side rooms. This demonstrates that rigorous droplet measures can contain the spread even in multi-occupancy wards when single rooms are unavailable [12].

Table 2 summarizes key hospital-based studies evaluating patient isolation and cohorting strategies during influenza outbreaks. Together, these reports demonstrate how targeted spatial interventions, ranging from single-room isolation to unit-wide cohorting, affect nosocomial transmission rates, resource utilization, and outbreak resolution. While methodologies varied, most studies reported measurable reductions in secondary cases or improved operational efficiency, underscoring the utility of structured placement strategies during hospital outbreaks.

Operationally, and based on commonly reported IPC practice, cohorting decisions should not be delayed pending influenza strain typing. When preliminary testing confirms influenza and cases are epidemiologically linked (e.g., same ward within a defined time window), temporary cohorting under the assumption of strain homogeneity is operationally appropriate. Strain or subtype confirmation rarely alters immediate infection-control actions and is typically used for surveillance rather than real-time bed management. If subsequent typing identifies discordant strains, cohorts can be re-evaluated, but early cohorting is critical to limit onward transmission.

#### 3.3.6. Personal Protective Equipment: Practice and Pitfalls

Standard plus droplet precautions should be maintained for the whole infectious period—seven days after symptom onset or 24 h after symptom resolution, whichever is longer—with surgical masks, eye protection during aerosol-generating procedures, and gowns and gloves when contamination is possible [3]. WHO-aligned guidance and regional IPC manuals emphasize the importance of wearing masks within one meter of the patient, practicing hand hygiene at every contact, and implementing “buddy-check” systems during donning and doffing to help reduce breaches [3,5]. Multicenter audits continue to identify doffing lapses as the most frequent failure, highlighting the need for visible cues at room entry and continual skills reinforcement [38].

However, real-world adherence to PPE and outbreak protocols is often limited by behavioral and organizational barriers, including risk perception gaps, PPE fatigue, inconsistent training, and variable institutional safety cultures. Studies have shown that multimodal staff education, clear role expectations, and real-time feedback can substantially improve compliance and effectiveness in outbreak control. Post-pandemic behavioral norms may also influence adherence to PPE. A recent study among university students in Taiwan found that voluntary mask wearing remained common in 2024–2025 and was strongly associated with interpersonal distancing and a lower perceived risk of infection [39]. These findings suggest that behavioral spillover effects from the COVID-19 era—particularly heightened risk awareness—may continue to reinforce mask use and other protective practices in healthcare settings.

#### 3.3.7. Active Case-Finding, Surveillance, and Exit Criteria

Once control measures are initiated, facilities should conduct daily active surveillance of all patients, staff, and essential visitors on affected units for fever, new cough, myalgia, or unexplained functional decline, with molecular testing for any symptomatic individuals [5]. Enhanced surveillance should continue for at least seven days after the last laboratory-confirmed case before declaring the outbreak over. Embedding symptom checks into routine vital-sign rounds enables earlier detection and isolation of secondary cases, thereby reducing onward transmission [3,5,12].

#### 3.3.8. Communication and Documentation

Efficient communication is integral to outbreak control. Clinicians should flag test orders as high priority to expedite laboratory processing and promptly relay positive results to the clinical team and IPC to trigger placement, treatment, admission restrictions, and enhanced environmental cleaning [5]. When influenza is ruled out and an alternate diagnosis is established, timely de-labeling of patients under investigation preserves isolation capacity and focuses resources where transmission risk is real.

#### 3.3.9. Clinician’s Role Across the Outbreak Lifecycle

Clinicians catalyze the response by recognizing and reporting index cases early and sustain control by adhering to testing algorithms, ensuring appropriate placement, enforcing PPE discipline, and initiating treatment and chemoprophylaxis per protocol [4,5]. Consistent protocol fidelity and vigilant surveillance curtail transmission, preserve workforce capacity, and protect high-risk populations, including the immunocompromised and older adults—who experience the greatest morbidity from nosocomial influenza [3,4,5,11,12,32,36,38].

### 3.4. Antiviral Therapy and Chemoprophylaxis

#### 3.4.1. Antiviral Therapy

Early neuraminidase-inhibitor therapy is central to inpatient influenza care. In >20,000 laboratory-confirmed cases, starting oseltamivir within 48 h of symptom onset or on the day of admission shortened length of stay by 1–2 days and reduced in-hospital mortality by 40–70% versus later or no treatment [40,41]; benefit persists to days 4–5 but is greatest with immediate therapy (aHR for death, 0.27; 95% CI, 0.13–0.55) [42]. Adverse effects are uncommon and mild; prescribe oseltamivir 75 mg twice daily (renal dose-adjusted) empirically when influenza is suspected, pending confirmation [43]. Table 3 summarizes 12 cohort and randomized studies showing consistent reductions in mortality, clinical failure, and length of stay with therapy initiated ≤48 h; retrospective cohorts report 18–64% relative reductions in 30-day mortality.

#### 3.4.2. Post-Exposure Prophylaxis

Post-exposure chemoprophylaxis (PEP) complements treatment during ward outbreaks. In three hospital investigations, oseltamivir 75 mg once daily started within 48 h of exposure for 3–5 days reduced secondary attack rates from 13 to 24% to ≤3% (relative risk reduction ≈ 82–100%), with <10% mild, transient gastrointestinal adverse effects [54,55,56]. A 2024 Lancet systematic review and network meta-analysis showed a ~60% reduction in laboratory-confirmed symptomatic influenza with the same regimen (RR 0.40, 95% CI 0.26–0.62) without a meaningful increase in adverse events [57]. A randomized non-inferiority trial found similar efficacy with 5- vs. 10-day courses (1.8% vs. 0%, *p* = 0.22), supporting 5 days for most exposures, with longer courses reserved for profoundly immunocompromised contacts or prolonged exposure windows [58]. Collectively, these data support rapid deployment of short-course PEP for exposed patients and staff—alongside empiric oseltamivir for suspected in-patient cases—to curb nosocomial transmission. Table 4 summarizes PEP studies in hospital outbreaks (typically 3–10 days of oseltamivir among exposed patients or staff).

### 3.5. Vaccination Strategies

#### 3.5.1. Integrating Influenza and COVID-19 Vaccination Strategies

Integrating influenza and COVID-19 vaccination strategies is now central to hospital influenza outbreak control, leveraging COVID-era infrastructure to deliver coordinated respiratory virus protection across settings [62,63]. Health systems can align vaccination with infection-prevention bundles and timely antivirals, standardize messaging for patients and staff (e.g., unified posters on respiratory precautions, scripted discharge instructions, and consistent outbreak bulletins reminding staff of PPE and testing requirements), and embed prompts into routine care pathways (e.g., inpatient discharges, antenatal and chronic-care clinics) to reduce missed opportunities [3,62,63]. Current guidance supports co-administration of influenza and COVID-19 vaccines in eligible populations, enabling unified delivery schedules and streamlined logistics [3].

#### 3.5.2. Healthcare Personnel Vaccination Coverage Trend

Healthcare personnel (HCP) vaccination remains the first line of defense to protect high-risk inpatients and maintain staffing resilience [3,63]. Yet recent surveillance shows erosion in coverage: in the United States 2023–2024 season, 75.4% of HCP received influenza vaccine overall, with notable variation by setting (acute care 89.1% vs. long-term care 65.2%) and role (pharmacists 93.9%, physicians 93.0%, nurses 87.6%, assistants/aides 63.2%); only 31.3% received the updated 2023–24 COVID-19 vaccine [64]. In Europe, median HCP influenza vaccine coverage was 22.1% in 2023–24, down from 25% in the prior season, underscoring persistent gaps and heterogeneity across countries [65]. Published surveys attribute this decline to vaccine fatigue, reduced risk perception, concerns regarding vaccine effectiveness, and persistent hesitancy among certain staff groups [66,67].

#### 3.5.3. Antiviral Chemoprophylaxis for Unvaccinated Personnel

During confirmed healthcare-associated influenza outbreaks, unvaccinated HCP may receive antiviral chemoprophylaxis to mitigate exposure risk and preserve work-force capacity; consideration also extends to any HCP when circulating strains are poorly matched to the vaccine [5]. For newly vaccinated HCP, short-term prophylaxis (up to two weeks) can bridge the period before vaccine-induced immunity develops, particularly in high-risk units or ongoing outbreaks [4].

#### 3.5.4. Patient Vaccination Integration Strategies

Post-COVID program design should embed influenza vaccination within broader respiratory-virus platforms that reuse COVID-19 delivery assets (sites, cold chain, data systems), apply standing orders and electronic prompts, and coordinate with outpatient and inpatient touchpoints to maximize uptake [62,63]. Co-administration policies and single-visit offerings simplify patient journeys [3]. Among staff-facing enablers, multicomponent, on-site, and role-tailored interventions (e.g., mobile carts, peer champions, convenient consent) consistently improve uptake and reduce missed opportunities [68].

#### 3.5.5. Vaccination as a Pillar of Workforce Resilience

Vaccination is a foundational—but not solitary—pillar of workforce resilience. Higher coverage, coupled with organizational support (adequate PPE, transparent IPC communication, continuous education, and routine health monitoring), is associated with better staff well-being and stability during surges [3,69,70]. Resilience programs should pair individual-level skills (e.g., emotion regulation, access to mental-health services) with institutional policies (robust sick leave, easy vaccine access within benefits), recognizing resilience as a shared responsibility between individuals and healthcare institutions [63,69,70].

In addition to structural enablers, behavioral norms shaped during the COVID-19 era continue to influence protective practices. Chen Y-L et al. reported that voluntary mask use remained common among university students in 2024–2025 and was closely linked to interpersonal distancing and precautionary attitudes [39]. Such behavioral spillover effects may similarly enhance adherence to vaccination, mask-wearing, and other respiratory virus precautions among healthcare personnel.

### 3.6. Communication and System-Level Coordination

In our conceptual framework (Figure 2), influenza outbreak management functions as an adaptive learning loop. Early detection triggers diagnostic escalation and isolation, thereby reducing the likelihood of onward transmission. Surveillance then feeds real-time information back to IPC teams, enabling adjustments in testing thresholds, PPE reinforcement, staff cohorting, or chemoprophylaxis. These iterative cycles not only shorten outbreak duration but also strengthen preparedness for subsequent seasons, aligning hospital responses with modern concepts of adaptive surveillance and policy-learning systems. Together, these clinical, epidemiological, and managerial feedback loops ensure that hospitals continuously refine their response as conditions evolve.

#### 3.6.1. Communication

Effective control requires disciplined, two-way communication. CDC guidance advises IPC teams to issue daily situation reports (including case counts, staffing, bed capacity, and control-measure reminders) to keep clinicians and administrators aligned [5]. Clinicians should document isolation status, oseltamivir start time and planned treatment/PEP duration in the electronic medical record (EMR) and reiterate these at every handover to enable real-time audit and timely de-isolation. Family discussions should explain the rationale for restrictions and request immediate reporting of any visitor’s respiratory symptoms.

#### 3.6.2. Visitor Management

Limit entry to essential visitors, screen all entrants for ILI, and enforce medical masking, particularly on geriatric/oncology wards [3,5]. Following the implementation of universal masking, observational data have shown significant reductions in healthcare-associated respiratory virus incidence [7,71,72]. Mitigate psychosocial costs by framing measures as time-limited and offering virtual visiting options, which improve acceptance [73].

#### 3.6.3. Unit or Service Modifications

**Figure 4:**
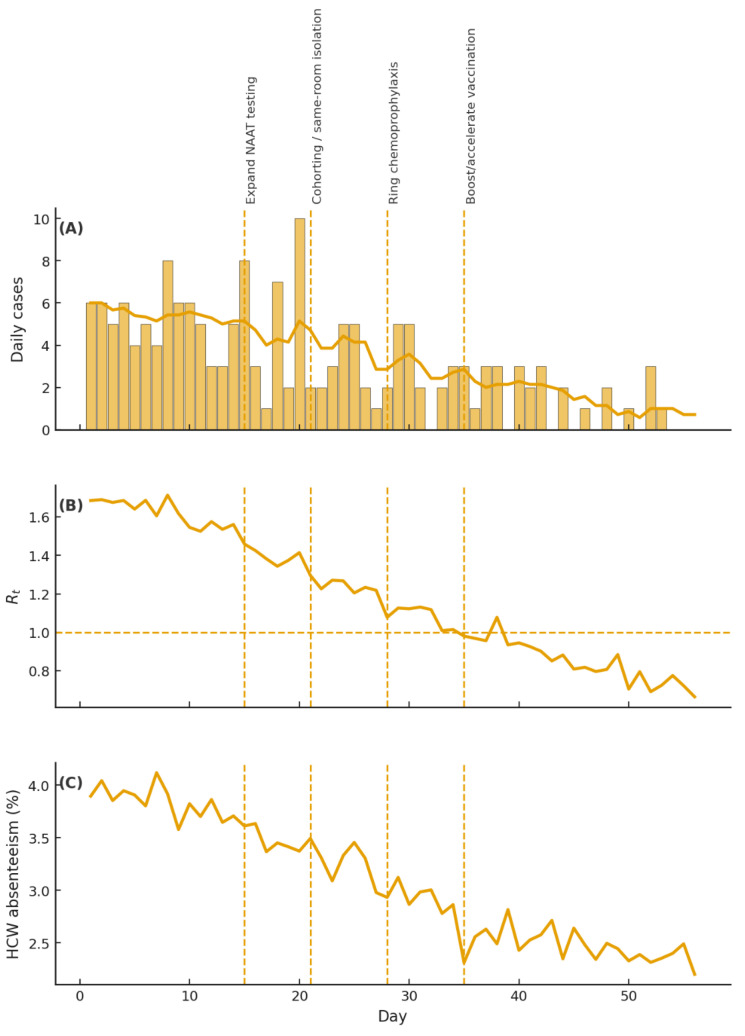
Interrupted time-series schematic of layered interventions. Simulated hospital outbreak metrics over 56 days showing: (**A**) daily influenza cases with a 7-day moving average; (**B**) time-varying effective reproduction number Rt (horizontal dashed line at Rt = 1.0); and (**C**) healthcare worker (HCW) absenteeism (%). Vertical dashed lines indicate the sequential control measures implemented during the observation period: expanding NAAT testing, cohorting/same-room isolation, ring chemoprophylaxis, and boosting/accelerating vaccination. The schematic illustrates the expected stepwise reduction in transmission (declining cases and Rt) and staffing impact (lower absenteeism) after each additional layer, consistent with a multi-component infection control response. Notes/Abbreviations: NAAT, nucleic acid amplification test; HCW, healthcare worker; Rt, effective reproduction number.

If transmission persists, escalate controls by temporarily closing affected wards to admissions/transfers, and deploying dedicated staff until at least 7 days after the last case [3]. During surges, defer elective surgery to decompress beds and reduce OR demand, thereby protecting capacity for acute respiratory admissions and reducing the backlog once the surge abates [74]. Peri-operative data show higher complication rates when influenza occurs within 2 weeks pre-operatively [75]. Utilize short-range forecasting tools (e.g., UK Health Security Agency (UKHSA)) to anticipate admission surges and inform decisions regarding ward closures or surgical deferrals [76]. As shown in Figure 4, the interrupted time series demonstrates stepwise changes in cases, Rt, and HCW absenteeism following each layered intervention.

For ease of reference, all practice statements and their corresponding evidence strength ratings are summarized in Table 5.

## 4. Discussion

This narrative review integrates guideline recommendations and post-COVID institutional experiences to synthesize contemporary strategies for controlling hospital influenza outbreaks. Across diverse study designs, several consistent themes emerged. Early detections, supported by rapid molecular diagnostics, remain central to interrupting transmission, enabling timely antiviral therapy, prompt isolation or cohorting, and the rapid activation of infection-control protocols. COVID-era practices, including broader N95 use during respiratory surges, improved ventilation, strengthened staff illness policies, and heightened adherence to PPE, have demonstrated measurable reductions in healthcare-associated respiratory viral infections and appear transferable to influenza control in many hospital environments. Significantly, population-level interventions implemented during the COVID-19 pandemic (e.g., community masking and mobility restrictions) primarily reduced influenza introduction into hospitals, whereas hospital-level measures—such as rapid molecular diagnostics, isolation, cohorting, and PPE policies—directly shaped in-hospital transmission dynamics and outbreak control. Importantly, the successful implementation of these measures depends on coordinated, multidisciplinary actions involving infection prevention and control teams, clinical microbiology laboratories, nursing leadership, hospital administration, and occupational health services, rather than on individual clinicians alone.

Despite widespread consensus among the WHO, CDC, and IDSA guidelines concerning fundamental principles, namely early detection, molecular diagnostics, prompt antiviral therapy, and isolation, the implementation of these recommendations exhibits considerable variation across different hospitals. Differences in infrastructure, laboratory capacity, staffing levels, and bed availability strongly influence how guidelines are implemented in practice. For example, recommendations for single-room isolation, rapid multiplex PCR testing, or extended staff cohorting may be readily achievable in tertiary medical centers but less feasible in smaller or resource-limited hospitals. Even in high-income settings, institutional thresholds for outbreak declaration, isolation duration, and post-exposure prophylaxis differ, reflecting local risk tolerance and operational constraints. These variations highlight the need to interpret guidelines as adaptable frameworks rather than rigid protocols, underscoring the importance of contextualized decision-making in hospital influenza outbreak management.

Interpretation of the available evidence should also consider key limitations of the sources. Much of the post-COVID literature on hospital influenza control is derived from descriptive outbreak reports, before–after studies, and institutional case series, with relatively few controlled or comparative evaluations. In addition, heterogeneity in outbreak definitions, intervention bundles, and reported outcomes limits direct cross-study comparison. These constraints indicate that many practice recommendations reflect pragmatic consensus and accumulated operational experience rather than high-certainty causal inference.

Resistance to neuraminidase inhibitors such as oseltamivir has been reported but remains uncommon globally (<1% in recent surveillance) [42]. Higher prevalence has been described in immunocompromised hosts and in cases of prolonged viral shedding, with some geographic variability. Ongoing WHO and CDC surveillance supports continued empiric oseltamivir use in hospital outbreak settings, while maintaining vigilance for treatment failure [77].

Despite their overall favorable risk–benefit profile, antiviral therapy and chemoprophylaxis are not without potential drawbacks. Neuraminidase inhibitors are generally well tolerated; however, gastrointestinal adverse effects, neuropsychiatric symptoms, and drug–drug interactions may reduce adherence, particularly among older adults and patients with multiple comorbidities. In addition, widespread or prolonged antiviral use during hospital outbreaks may impose selective pressure that facilitates the emergence of resistance, especially in immunocompromised patients with prolonged viral shedding. From an operational perspective, large-scale chemoprophylaxis can also strain pharmacy services, medication supply chains, and clinical workflows, underscoring the importance of targeted, time-limited antiviral strategies guided by risk stratification and ongoing surveillance rather than indiscriminate use.

However, the feasibility and sustainability of these measures vary substantially across healthcare systems. High-income hospitals often have access to negative-pressure rooms, on-site molecular testing platforms, respiratory protection programs, and dedicated infection prevention personnel. In contrast, resource-limited settings may face infrastructural constraints, inconsistent PPE supplies, limited laboratory capacity, and workforce shortages, all of which diminish the ability to operationalize layered precautions [78].

Because most available data originate from high-resource hospitals, the applicability of certain post-COVID practices to resource-limited settings requires careful consideration. Constraints in molecular diagnostic capacity, isolation infrastructure, staffing, and antiviral availability may necessitate alternative approaches, such as syndromic surveillance, simplified cohorting strategies, and prioritization of high-risk units rather than hospital-wide interventions.

Cost-effectiveness and long-term sustainability are critical considerations for post-COVID influenza control strategies. Resource-intensive measures—such as universal N95 use, rapid multiplex molecular testing, prolonged isolation, and dedicated staff cohorting—may generate substantial operational costs and workforce burden if maintained indiscriminately across extended influenza seasons. In contrast, targeted deployment of layered interventions, focused on high-risk units, peak transmission periods, or time-limited outbreak responses, may preserve clinical effectiveness while improving economic and operational sustainability. Balancing the incremental benefit of each intervention against its resource demands will be essential for informing durable hospital infection-control policies.

During seasonal surges, even well-resourced hospitals may struggle to sustain single-room isolation and dedicated cohorting because of bed pressures and staffing demands. Variations in healthcare worker vaccination coverage, antiviral stockpiles, and access to multiplex PCR further contribute to heterogeneity in outbreak response. These disparities highlight the need for contextual adaptation rather than uniform application of post-COVID protocols. Fundamental principles—rapid detection, timely diagnostics, early antiviral therapy, and protection of healthcare personnel—remain universal; however, their implementation must be tailored to local resources, infrastructure, and operational realities.

There are some limitations in this review. Reports from low-and middle-income countries (LMICs) are limited, and few studies rigorously evaluate the real-world effectiveness or cost-effectiveness of layered control strategies outside high-income hospitals. Similarly, the heterogeneity of outbreak investigations precludes meta-analytic pooling, and important questions remain regarding the optimal balance between diagnostic intensity, antiviral use, and isolation practices during high-burden seasons.

Looking ahead, several emerging tools may reshape hospital influenza control. Novel antivirals, rapid multiplex molecular assays, digital surveillance platforms, and AI-assisted early warning systems offer promising opportunities to strengthen outbreak detection and accelerate responses. Future research should evaluate the implementation, scalability, and clinical impact of these technologies across diverse hospital settings, particularly in LMICs. Understanding how to tailor multi-layered outbreak control bundles to local constraints will be essential for building resilient and equitable respiratory virus preparedness frameworks.

## 5. Conclusions

**Figure 5:**
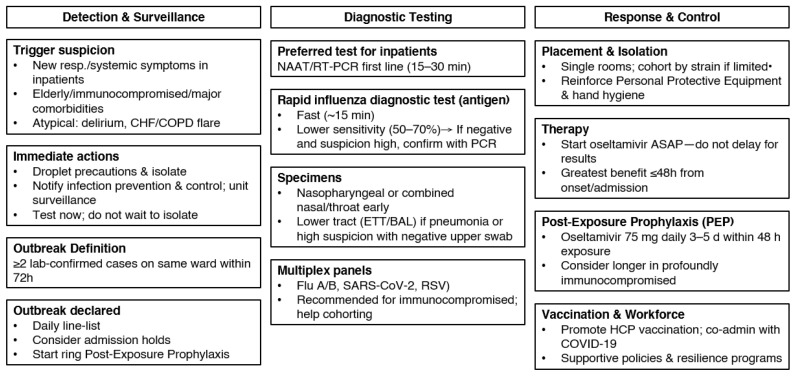
Management of influenza outbreak in hospitals.

This narrative review synthesizes multi-source guideline recommendations and post-COVID institutional experiences to present an integrated, system-level framework for detecting and managing hospital influenza outbreaks. Influenza outbreaks in hospitals require prompt, coordinated responses that extend beyond individual clinical actions and are grounded in surveillance, infection prevention, operational planning, and institutional governance. Figure 5 summarizes these core response domains, showing how transmission precautions, infection-control policies, diagnostic strategies, and early detection & surveillance converge on the shared goal of preventing a hospital-wide outbreak.

Early recognition, even of a single nosocomial case, is essential, with two linked cases typically prompting the declaration of an outbreak. Effective control depends on rapid, multi-layered interventions: isolation of suspected cases, prompt molecular testing, and early initiation of antiviral therapy without waiting for confirmation. Starting antiviral therapy within 48 h of symptom onset consistently reduces severity, mortality, and hospital stay. Infection-control measures, enhanced droplet or aerosol precautions, patient cohorting, ward restrictions, and PPE use are critical, especially in consideration of lessons from the COVID-19 era. Post-exposure prophylaxis for exposed individuals and daily active surveillance further reduce transmission risk.

Significantly, the success of these measures relies on coordinated, multidisciplinary collaboration among infection prevention and control teams, clinical microbiology laboratories, nursing leadership, hospital administration, occupational health services, and frontline clinicians. Within this coordinated framework, clinicians play a crucial but non-exclusive role by recognizing cases, initiating appropriate diagnostics and treatment, and adhering to institutional outbreak protocols. Taken together, these findings underscore that hospital influenza outbreak management is fundamentally a system-level intervention. Embedding these evidence-informed, multidisciplinary strategies into routine seasonal preparedness is essential for strengthening institutional resilience and protecting high-risk patient populations.

Future efforts should focus on evaluating the effectiveness, feasibility, and sustainability of multidisciplinary outbreak protocols across diverse hospital settings, and on updating institutional guidelines as diagnostic technologies, antiviral strategies, and respiratory virus epidemiology continue to evolve.

## Figures and Tables

**Table 1:** Pre- vs. Post-COVID Hospital Influenza Response.

Aspect	Pre-COVID (Traditional)	Post-COVID Observations
Transmission	Primary transmission occurred via droplet and contact routes, with airborne precautions applied only during aerosol-generating procedures (AGPs) [10]. Hospitals typically relied on standard droplet precautions using surgical masks.	The potential for airborne transmission became more emphasized, and N95 respirators were routinely used during AGPs. Many facilities implemented ventilation improvements (e.g., HEPA filtration), and strict non-pharmaceutical interventions, such as universal masking and social distancing, were shown to reduce influenza spread substantially [8,9].
Clinical Features	Typical symptoms included abrupt onset of fever, cough, and myalgia [10]. Older adults more often presented atypically (e.g., confusion), and children occasionally exhibited gastrointestinal symptoms.	Clinical features increasingly overlapped with COVID-19 and RSV, complicating syndromic diagnosis. Coinfections also became more common [13], and multiplex PCR was more widely adopted to differentiate respiratory pathogens.
Incubation & Infectivity	The incubation period was typically 1–4 days, and patients were infectious from 1 day before to 5–7 days after symptom onset; children and immunocompromised individuals could shed virus for longer durations [10].	The biologic characteristics of infectivity remained unchanged, but control practices became stricter. Patients were isolated until symptom resolution or for at least 7 days [11], with more conservative sick-leave policies and earlier testing and quarantine of contacts.

**Table 2:** Effectiveness of Patient Isolation or Cohorting during Hospital Influenza Outbreaks.

Study (Year) [Ref.]	Design	Sample Size (n)	Intervention/Strategy	Nosocomial Outcome †	Operational/Clinical Impact ‡
Adal 1996 [33]	Retrospective before–after	NR	Isolation + staff furlough	No significant change	NR
Sartor 2002 [34]	Prospective cohort	23 patients/22 staff	Isolation (details NR)	Attack rate: 41% (patients); 23% (staff)	Cost ↑ USD 34k; ED closure 11 days
Salgado 2004 [35]	Prospective before–after	NR	Mobile vaccination cart	↓ incidence (*p* < 0.0001)	NR
Munier-Marion 2016 [36]	Prospective cohort	NR	Single-room vs. double-room occupancy	0.7 vs. 2.0 cases/100 patient-days (*p* = 0.028)	NR
Youngs 2019 [11]	Prospective before–after	654 patients	Point-of-care testing + cohort ward	↓ cases/day 0.95 → 0.66 (*p* < 0.0001)	LOS ↓ 2.0 days; 779 single-room bed-days released
Suh 2023 [37]	Retrospective cohort	814 children	Admission isolation and cohorting	Secondary attack rate 1%; hospital-acquired flu 3%	NR
Birrer 2024 [12]	Prospective cohort	764 at-risk patients	DroPS (in-room droplet precautions)	HARVI 0.4% (flu/RSV)	Single-room capacity preserved

† Primary transmission metric as reported by each study. Arrows indicate direction of change (↓ decrease/↑ increase). ‡ Includes length of stay (LOS), bed-days, cost, staff absenteeism, or service disruption. NR = not reported; HARVI = healthcare-associated respiratory viral infection.

**Table 3:** Effectiveness of Antiviral Therapy during Hospital Influenza Outbreaks.

Study (Year) [Refs.]	Design	Sample Size (n)	Population	Early Treatment Definition *	LOS Change †	Adjusted Effect ‡
Lee 2007 [44]	Retrospective cohort	356	Adults	≤48 h from symptom onset	↓ 2 days	aHR 0.65 (0.52–0.81) mortality
Lee 2010 [45]	Prospective cohort	754	Adults	≤48 h from symptom onset	Earlier discharge	aHR 0.78 (0.64–0.96) mortality
Ebell 2013 [46]	Systematic review (RCTs)	4327	Adults	≤36 h from symptom onset	↓ symptom duration 17 h	Effect size NR
Ison 2013 [47]	Randomized controlled trial	137	Adults	≤48 h from symptom onset	NR	HR 0.84 (CI NR) faster clinical stability
Ramirez 2018 [48]	Randomized controlled trial	1107	Adults	≤48 h from symptom onset	NR	RR 0.74 (0.60–0.92) clinical failure
Dou 2020 [40]	Single-center retrospective cohort	433	Adults	≤8 h from symptom onset	↓ 1.3 days	Effect size NR
Groeneveld 2020 [49]	Multicenter retrospective cohort	390	Adults	≤48 h from admission	NR	aHR 0.60 (CI NR) 30-day mortality
Sharma 2021 [50]	Multicenter retrospective cohort	1828	Adults	≤48 h from admission	↓ LOS (value NR)	aHR 0.54 (0.35–0.84) readmission/mortality
Wiemken 2021 [51]	Secondary analysis of RCT	691	Adults	≤48 h from symptom onset	NR	RR 0.54 (0.34–0.86) clinical failure
Walsh 2022 [52]	Multicenter pediatric cohort	11,294	Children	≤24 h from admission	↓ 0.6 day	OR 0.46 (0.32–0.68) §
Lewis 2024 [41]	Prospective multicenter cohort	840	Adults	Admission Day 0 (≤24 h from admission)	NR	aOR 0.36 (0.18–0.72) mortality
Pott 2025 [53]	Pooled cohort (75 hospitals)	8135	Adults	≤48 h from admission	NR	aHR 0.82 (0.69–0.98) 30-day mortality

* Early treatment definitions were reproduced from original studies and harmonized as hours from symptom onset or from hospital admission. † LOS = length of stay; “↓” indicates shortened LOS; NR = not reported. ‡ Adjusted effect includes main adjusted estimates (aHR, aOR, RR, HR) comparing early vs. later/no therapy; 95% CI in parentheses. § Composite outcome (ICU/ECMO/death); reference ≥ 24 h after admission.

**Table 4:** Effectiveness of Post-Exposure Prophylaxis (PEP) during Hospital Influenza Outbreaks.

Study (Year) [Refs.]	Design	Setting	Sample (n)	PEP Regimen (Duration)	Secondary Infection Rate *	Protection/Outcome
Shinjoh 2004 [59]	Prospective	Pediatric wards	29	Oseltamivir 7–10 days	0% (PEP) vs. 69% (no-PEP)	100% protection
Vu 2007 [60]	Case–control	HSCT outpatient facility	45	Oseltamivir 10–81 days (median 17)	NR	Outcome NR
Shinjoh 2012 [61]	Retrospective	Pediatric wards	81	Oseltamivir or Zanamivir (duration NR)	3% (PEP) vs. 29% (no-PEP)	90% protection
Ishiguro 2016 [54]	Observational cohort	Hospital ward	NR	Oseltamivir 3 days	1.0% (PEP) vs. 16.7% (no-PEP)	93% protection
Lepen 2020 [58]	Open-label RCT	University hospital	222	Oseltamivir 5 days vs. 10 days	1.8% (5 d) vs. 0% (10 d)	5-day regimen non-inferior
Wrotek 2024 [55]	Randomized open-label pilot	Pediatric wards	59	Oseltamivir 3 days vs. 7 days	0% (3 d) vs. 6.4% (7 d)	3-day regimen non-inferior; cost ↓

* Secondary infection is defined as laboratory-confirmed influenza among contacts after PEP initiation. Comparators include no-PEP or alternative-duration groups. NR = not reported; HSCT = hematopoietic stem-cell transplant. Protection (%) reflects (1—secondary attack risk ratio), where applicable. ↓ Indicates decrease.

**Table 5:** Summary of Practice Statements for Hospital Influenza Outbreak Management.

No	Practice Statement (Concise)	Evidence Strength *
1	Maintain a high index of suspicion for influenza in hospitalized patients with new respiratory symptoms or atypical presentations (e.g., delirium in older adults), especially during influenza season.	High
2	Use nucleic acid amplification tests (NAATs) as the preferred diagnostic modality for hospitalized patients; negative antigen tests should be confirmed with PCR.	High
3	In severe disease or pneumonia, obtain lower respiratory tract specimens when upper respiratory samples are negative but clinical suspicion remains high.	Moderate
4	Define a hospital influenza outbreak as ≥2 epidemiologically linked, laboratory-confirmed cases within 72 h on the same ward to trigger outbreak response measures.	Moderate
5	Initiate isolation or cohorting immediately upon suspicion or confirmation of influenza; do not delay placement decisions pending strain or subtype confirmation.	High
6	Apply layered infection-control measures during outbreaks, including droplet precautions, appropriate respiratory protection, ventilation optimization, and dedicated staff cohorting where feasible.	Moderate
7	Start empiric oseltamivir as early as possible (ideally ≤48 h from symptom onset or on admission) in hospitalized patients with suspected or confirmed influenza.	High
8	Do not delay antiviral therapy while awaiting diagnostic confirmation in critically ill or high-risk patients.	High
9	Implement short-course post-exposure antiviral prophylaxis (PEP) for exposed patients and healthcare workers during confirmed ward outbreaks.	High
10	Conduct daily active surveillance for new symptoms among patients and relevant staff on affected units until at least 7 days after the last confirmed case.	Moderate
11	Integrate influenza vaccination strategies for patients and healthcare personnel into routine hospital preparedness to reduce outbreak risk and workforce disruption.	High
12	Ensure timely communication, documentation, and coordination among clinicians, infection-prevention teams, and hospital leadership throughout the outbreak lifecycle.	Moderate

* Evidence strength reflects convergence of guideline recommendations, consistency of observational and interventional studies, and applicability to adult inpatient settings (classified as High or Moderate).

## Data Availability

No new data were created or analyzed in this study. Data sharing is not applicable to this article.

## References

[1] World Health Organization (2025). Influenza (Seasonal). https://www.who.int/news-room/fact-sheets/detail/influenza-(seasonal).

[2] Centers for Disease Control and Prevention (2024). About Estimated Flu Burden. https://www.cdc.gov/flu-burden/php/about/.

[3] Centers for Disease Control and Prevention (2025). Infection Prevention and Control Strategies for Seasonal Influenza in Healthcare Settings. https://www.cdc.gov/flu/professionals/infectioncontrol.

[4] Uyeki T.M., Bernstein H.H., Bradley J.S., Englund J.A., File T.M., Fry A.M., Pavia A.T. (2019). Clinical Practice Guidelines by the Infectious Diseases Society of America: 2018 Update on Diagnosis, Treatment, Chemoprophylaxis, and Institutional Outbreak Management of Seasonal Influenza. Clin. Infect. Dis..

[5] Centers for Disease Control and Prevention (2024). Interim Guidance for Influenza Outbreak Management in Long-Term Care and Post-Acute Care Facilities. https://www.cdc.gov/flu/hcp/infection-control/ltc-facility-guidance.html.

[6] World Health Organization (2024). Clinical Practice Guidelines for Influenza. https://www.who.int/publications/i/item/9789240097759.

[7] Yan J., McClure T., Aslam A., Bubb T., Babady N.E., Usiak S., Kamboj M. (2024). Impact of Universal Masking in Reducing the Risk of Nosocomial Respiratory Viruses among People with Cancer. Infect. Control Hosp. Epidemiol..

[8] Olsen S.J. (2020). Decreased Influenza Activity during the COVID-19 Pandemic—United States, Australia, Chile, and South Africa, 2020. MMWR Morb. Mortal. Wkly. Rep..

[9] Chan L.Y.H., Morris S.E., Stockwell M.S., Bowman N.M., Asturias E., Rao S., Lutrick K., Ellingson K.D., Nguyen H.Q., Maldonado Y. (2024). Estimating the generation time for influenza transmission using household data in the United States. medRxiv.

[10] Baek J.H., Seo Y.B., Choi W.S., Kee S.Y., Jeong H.W., Lee H.Y., Kim W.J. (2014). Guideline on the Prevention and Control of Seasonal Influenza in Healthcare Settings. Korean J. Intern. Med..

[11] Youngs J., Marshall B., Farragher M., Knapper F., McGrath E., Riley P., Shallcross L., Edgeworth J.D. (2019). Implementation of Influenza Point-of-Care Testing and Patient Cohorting during a High-Incidence Season: A Retrospective Analysis of Impact on Infection Prevention and Control and Clinical Outcomes. J. Hosp. Infect..

[12] Birrer M., Draps K., Hobi F., Guery B., Manuel O., Widmer A., Sax H. (2024). Hospital-Acquired Respiratory Viral Infections While Applying Droplet Precautions On Site (DroPS): Prospective Observation during the 2019/20 Influenza Season, Bern, Switzerland. Infect. Prev. Pract..

[13] Pratt G.W., Wong C.L., Rao L.V. (2025). Prevalence and Co-Detection Rates of SARS-CoV-2, Influenza, and Respiratory Syncytial Virus: A Retrospective Analysis. APMIS.

[14] Clark T.W., Lindsley K., Wigmosta T.B., Bhagat A., Hemmert R.B., Uyei J., Timbrook T.T. (2023). Rapid Multiplex PCR for Respiratory Viruses Reduces Time to Result and Improves Clinical Care: Results of a Systematic Review and Meta-analysis. J. Infect..

[15] Verdier V., Lilienthal F., Desvergez A., Gazaille V., Winer A., Paganin F. (2023). Severe Forms of Influenza Infections Admitted in Intensive Care Units: Analysis of Mortality Factors. Influenza Other Respir. Viruses.

[16] Bilgin H., Başarı T., Pazar N., Küçüker I., Can-Sarınoğlu R. (2023). Comparison of 28-Day Mortality between Hospital- and Community-Acquired Influenza Patients. Infect. Dis. Clin. Microbiol..

[17] Centers for Disease Control and Prevention (2024). Clinical Signs and Symptoms of Influenza. https://www.cdc.gov/flu/hcp/clinical-signs/index.html.

[18] Falsey A.R. (2025). Neurologic Complications of Influenza and Potential Protective Vaccine Effects. Influenza Other Respir. Viruses.

[19] Huang Y., Li S., Ye W., Wang H., Su J., Gao L., Chen G. (2025). Viral Infections in Elderly Individuals: A Comprehensive Overview of SARS-CoV-2 and Influenza Susceptibility, Pathogenesis, and Clinical Treatment Strategies. Vaccines.

[20] Merckx J., Wali R., Schiller I., Caya C., Gore G.C., Chartrand C., Papenburg J. (2017). Diagnostic Accuracy of Novel and Traditional Rapid Tests for Influenza Infection Compared with Reverse Transcriptase Polymerase Chain Reaction: A Systematic Review and Meta-analysis. Ann. Intern. Med..

[21] Centers for Disease Control and Prevention Rapid Influenza Diagnostic Tests. https://www.cdc.gov/flu/hcp/testing-methods/clinician_guidance_ridt.html.

[22] Berry G.J., Jhaveri T.A., Larkin P.M., Mostafa H., Babady N.E. (2024). ADLM Guidance Document on Laboratory Diagnosis of Respiratory Viruses. J. Appl. Lab. Med..

[23] Centers for Disease Control and Prevention Guide for Considering Influenza Testing When Influenza Viruses Are Circulating in the Community. https://www.cdc.gov/flu/hcp/testing-methods/consider-influenza-testing.html.

[24] Yin H., Wu W., Lv Y., Kou H., Sun Y. (2025). Comparative Evaluation of Three Rapid Influenza Diagnostic Tests for Detection of Influenza A and B Viruses Using RT-PCR as the Reference Method. J. Med. Virol..

[25] Sanz-Muñoz I., Sánchez-Martínez J., Rodríguez-Crespo C., Arroyo-Hernantes I., Domínguez-Gil M., Rojo Rello S., Eiros J.M. (2024). Association of Viral Loads of Influenza A (H3N2) with Age and Care Setting on Presentation: A Prospective Study during the 2022–2023 Influenza Season in Spain. Int. J. Infect. Dis..

[26] Chan W.S., Ho C.W.Y., Chan T.C., Hung J., To M.Y., Leung S.M., Tang B.S.F. (2024). Clinical Evaluation of the BIOFIRE SPOTFIRE Respiratory Panel. Viruses.

[27] Su X., Zhou J., Liu L., Gao H., Lin Y., Wang Z., Guo W. (2024). Performance Evaluation of Influenza A Rapid Antigen Test and PCR among Nasopharyngeal and Oropharyngeal Samples. Practic. Lab. Med..

[28] Zhang C., Fang V.J., Chan K.H., Leung G.M., Ip D.K., Peiris J.M., Tsang T.K. (2025). Interplay between Viral Shedding, Age, and Symptoms in Individual Infectiousness of Influenza Cases in Households. J. Infect. Dis..

[29] Ramirez J., Furmanek S., Chandler T., Carrico R., Wilde A., Junkins A., Raghuram A. (2025). Diagnosis of Community-Acquired Pneumonia due to Influenza or Respiratory Syncytial Virus: Evaluation of RT-PCR Sensitivity in Nasopharyngeal, Saliva, and Sputum Samples. Pathogens.

[30] Centers for Disease Control and Prevention Clinical Guidance for Hospitalized and Non-Hospitalized Patients When SARS-CoV-2 and Influenza Viruses Are Co-Circulating. https://www.cdc.gov/flu/hcp/clinical-guidance/testing-guidance-for-clinicians.html.

[31] Liu Y., Wang Y., Mai H., Chen Y., Liu B., Liu Y., Gao Y. (2022). Clinical Characteristics, Risk Factors, and Antiviral Treatments of Influenza in Immunosuppressed Inpatients in Beijing during the 2015–2020 Influenza Seasons. Virol. J..

[32] World Health Organization (2024). Implementing the Integrated Sentinel Surveillance of Influenza and Other Respiratory Viruses of Epidemic and Pandemic Potential by the Global Influenza Surveillance and Response System: Standards and Operational Guidance. https://www.who.int/publications/i/item/9789240101432.

[33] Adal K.A., Flowers R.H., Anglim A.M., Hayden F.G., Titus M.G., Coyner B.J., Farr B.M. (1996). Prevention of Nosocomial Influenza. Infect. Control Hosp. Epidemiol..

[34] Sartor C., Zandotti C., Romain F., Jacomo V., Simon S., Atlan-Gepner C., Drancourt M. (2002). Disruption of Services in an Internal Medicine Unit Due to a Nosocomial Influenza Outbreak. Infect. Control Hosp. Epidemiol..

[35] Salgado C.D., Giannetta E.T., Hayden F.G., Farr B.M. (2004). Preventing Nosocomial Influenza by Improving the Vaccine Acceptance Rate of Clinicians. Infect. Control Hosp. Epidemiol..

[36] Munier-Marion E., Bénet T., Régis C., Estellat C., Morey J., Jacquemoud M., Timsit J.F., Vanhems P. (2016). Hospitalization in Double-Occupancy Rooms and the Risk of Hospital-Acquired Influenza: A Prospective Cohort Study. Clin. Microbiol. Infect..

[37] Suh W., Han S.B. (2023). Nosocomial Influenza in a Pediatric General Ward: Effects of Isolation and Cohort Placement of Children with Influenza. Infect. Control Hosp. Epidemiol..

[38] Ohishi T., Shinomiya S., Tsuda Y., Kurosu H., Yoshikawa T. (2025). Personal Protective Equipment Stewardship across 112 Medical Facilities: A Preliminary Survey in Japan. J. Hosp. Infect..

[39] Chen Y.-L., Lin M.-C., Chang Y.-C., Hsiao Y.-T., Lin Y.-H., Wu P.-Y., Lee M.-C. (2025). From Mandate to Choice: How Voluntary Mask Wearing Shapes Interpersonal Distance among University Students after COVID-19. Healthcare.

[40] Dou L., Reynolds D., Wallace L., O’Horo J., Kashyap R., Gajic O., Yadav H. (2020). Decreased Hospital Length of Stay with Early Administration of Oseltamivir in Patients Hospitalized with Influenza. Mayo Clin. Proc. Innov. Qual. Outcomes.

[41] Lewis N.M., Harker E.J., Grant L.B., Zhu Y., Grijalva C.G., Chappell J.D., Self W.H. (2024). Benefit of Early Oseltamivir Therapy for Adults Hospitalized with Influenza A: An Observational Study. Clin. Infect. Dis..

[42] Adlhoch C., Delgado-Sanz C., Carnahan A., Larrauri A., Popovici O., Bossuyt N., Olsen S.J. (2023). Effect of Neuraminidase-Inhibitor (Oseltamivir) Treatment on Outcomes of Hospitalised Influenza Patients: Surveillance Data from 11 EU Countries, 2010–2020. Euro Surveill..

[43] Gao Y., Guyatt G., Uyeki T.M., Liu M., Chen Y., Zhao Y., Hao Q. (2024). Antivirals for Treatment of Severe Influenza: A Systematic Review and Network Meta-analysis of Randomised Controlled Trials. Lancet.

[44] Lee N., Chan P.K., Choi K.W., Lui G., Wong B., Cockram C.S., Sung J.J. (2007). Factors Associated with Early Hospital Discharge of Adult Influenza Patients. Antivir. Ther..

[45] Lee N., Choi K.W., Chan P.K.S., Hui D.S.C., Lui G.C.Y., Wong B.C.K., Sung J.J.Y. (2010). Outcomes of Adults Hospitalised with Severe Influenza. Thorax.

[46] Ebell M.H., Call M., Shinholser J. (2013). Effectiveness of Oseltamivir in Adults: A Meta-analysis of Published and Unpublished Clinical Trials. Fam. Pract..

[47] Ison M.G., Hui D.S., Clezy K., O’Neil B.J., Flynt A., Collis P.J., Alexander W.J. (2013). A Clinical Trial of Intravenous Peramivir Compared with Oral Oseltamivir for the Treatment of Seasonal Influenza in Hospitalized Adults. Antivir. Ther..

[48] Ramirez J., Peyrani P., Wiemken T., Chaves S.S., Fry A.M., Arnold F.W. (2018). A Randomized Study Evaluating the Effectiveness of Oseltamivir Initiated at the Time of Hospital Admission in Adults Hospitalized with Influenza-Associated Lower Respiratory Tract Infections. Clin. Infect. Dis..

[49] Groeneveld G.H., Marbus S.D., Ismail N., de Vries J.J., Schneeberger P., Oosterheert J.J., de Boer M.G. (2020). Effectiveness of Oseltamivir in Reduction of Complications and 30-Day Mortality in Severe Seasonal Influenza Infection. Int. J. Antimicrob. Agents.

[50] Sharma Y., Horwood C., Hakendorf P., Thompson C. (2021). Effectiveness of Oseltamivir in Reducing 30-Day Readmissions and Mortality among Patients with Severe Seasonal Influenza in Australian Hospitalized Patients. Int. J. Infect. Dis..

[51] Wiemken T.L., Furmanek S.P., Carrico R.M., Peyrani P., Hoft D., Fry A.M., Ramirez J.A. (2021). Effectiveness of Oseltamivir Treatment on Clinical Failure in Hospitalized Patients with Lower Respiratory Tract Infection. BMC Infect. Dis..

[52] Walsh P.S., Schnadower D., Zhang Y., Ramgopal S., Shah S.S., Wilson P.M. (2022). Association of Early Oseltamivir with Improved Outcomes in Hospitalized Children with Influenza, 2007–2020. JAMA Pediatr..

[53] Pott H., Andrew M.K., Shaffelburg Z., Nichols M.K., Ye L., ElSherif M., McNeil S.A. (2025). Oseltamivir Reduces 30-Day Mortality in Older Adults with Influenza: A Pooled Analysis from the 2012–2019 Serious Outcomes Surveillance Network of the Canadian Immunization Research Network. Open Forum Infect. Dis..

[54] Ishiguro N., Oyamada R., Nasuhara Y., Yamada T., Miyamoto T., Imai S., Ono K. (2016). Three-Day Regimen of Oseltamivir for Post-Exposure Prophylaxis of Influenza in Wards. J. Hosp. Infect..

[55] Wrotek A., Jackowska T. (2024). A Non-Inferiority Randomized Open-Label Pilot Study of 3- versus 7-Day Influenza Post-Exposure Prophylaxis with Oseltamivir in Hospitalized Children. Sci. Rep..

[56] Dronavalli M., Lord H., Alexander K., Boonwaat L., Pal N., Fletcher-Lartey S.M. (2020). Effectiveness of Oseltamivir Prophylaxis in Influenza Outbreaks in Residential Aged Care. J. Epidemiol. Glob. Health.

[57] Zhao Y., Gao Y., Guyatt G., Uyeki T.M., Liu P., Liu M., Hao Q. (2024). Antivirals for Post-Exposure Prophylaxis of Influenza: A Systematic Review and Network Meta-Analysis. Lancet.

[58] Lepen L., Blagus R., Velušček M., Saletinger R., Petrovec M., Bajrović F.F., Stupica D. (2020). Five-Day versus 10-Day Post-Exposure Chemoprophylaxis with Oseltamivir to Prevent Hospital Transmission of Influenza: A Non-Inferiority Randomized Open-Label Study. Open Forum Infect. Dis..

[59] Shinjoh M., Sato S., Sugaya N., Mitamura K., Takeuchi Y., Kosaki K., Takahashi T. (2004). Effect of Post-Exposure Prophylaxis with Oseltamivir for Those in Contact with Influenza Patients in Pediatric Wards. Kansenshogaku Zasshi.

[60] Vu D., Peck A.J., Nichols W.G., Varley C., Englund J.A., Corey L., Boeckh M. (2007). Safety and Tolerability of Oseltamivir Prophylaxis in Hematopoietic Stem Cell Transplant Recipients: A Retrospective Case-Control Study. Clin. Infect. Dis..

[61] Shinjoh M., Takano Y., Takahashi T., Hasegawa N., Iwata S., Sugaya N. (2012). Post-Exposure Prophylaxis for Influenza in Pediatric Wards: Oseltamivir or Zanamivir after Rapid Antigen Detection. Pediatr. Infect. Dis. J..

[62] World Health Organization (2022). Considerations for Integrating COVID-19 Vaccination into Immunization Programmes and Primary Health Care for 2022 and Beyond.

[63] National Academy of Medicine (NAM) (2023). The Influenza Imperative. *NAM Perspect*. https://nam.edu/wp-content/uploads/2022/09/The-Influenza-Imperative_final.pdf.

[64] Bell J., Meng L., Barbre K., Wong E., Lape-Newman B., Koech W., Thompson N.D. (2024). Influenza and COVID-19 Vaccination Coverage among Health Care Personnel—National Healthcare Safety Network, United States, 2023–2024Respiratory Virus Season. MMWR Morb. Mortal. Wkly. Rep..

[65] European Centre for Disease Prevention and Control (ECDC) (2024). Survey Report on National Seasonal Influenza Vaccination Recommendations and Vaccination Coverage Rates in the EU/EEA, 2023–2024 Season.

[66] Challenger A., Sumner P., Powell E., Bott L. (2023). Identifying Reasons for Non-Acceptance of Influenza Vaccine in Healthcare Workers: An Observational Study Using Declination Form Data. BMC Health Serv. Res..

[67] Guillari A., Polito F., Pucciarelli G., Serra N., Gargiulo G., Esposito M.R., Simeone S. (2021). Influenza Vaccination and Healthcare Workers: Barriers and Predisposing Factors—A Literature Review. Acta Biomed..

[68] de Koning R., Gonzalez Utrilla M., Spanaus E., Moore M., Lomazzi M. (2024). Strategies Used to Improve Vaccine Uptake among Healthcare Providers: A Systematic Review. Vaccine X.

[69] Curtin M., Corrigan P., Elshami A., Naughton C., Connolly R. (2022). Resilience among Health Care Workers during a Pandemic: A Meta-Synthesis. Clin. Psychol. Rev..

[70] Baskin R.G., Bartlett R. (2021). Healthcare Worker Resilience during COVID-19: An Integrative Review. J. Nurs. Manag..

[71] Pak T.R., Chen T., Kanjilal S., McKenna C.S., Rhee C., Klompas M. (2024). Testing and Masking Policies and Hospital-Onset Respiratory Viral Infections. JAMA Netw. Open.

[72] MacIntyre C.R., Chughtai A.A., Kunasekaran M., Tawfiq E., Greenhalgh T. (2025). The Role of Masks and Respirators in Preventing Respiratory Infections in Healthcare and Community Settings. BMJ.

[73] Holdsworth L.M., Siden R., Wong B.O., Verano M., Lessios A.S., Tabor H.K., Aslakson R. (2024). “Like Not Having an Arm”: A Qualitative Study of the Impact of Visitor Restrictions on Cancer Care during the COVID-19 Pandemic. Support. Care Cancer.

[74] Matias M.A., Santos R., Gutacker N., Mason A., Rice N. (2025). What Can We Learn about the Impact of Cancelled Planned Operations on Waiting Times? A Case Study Using the 2017/18 Winter Flu Postponement Policy in England. Health Econ. Rev..

[75] Lam F., Liao C.C., Chen T.L., Huang Y.M., Lee Y.J., Chiou H.Y. (2023). Outcomes after Surgery in Patients with and without Recent Influenza: A Nationwide Population-Based Study. Front. Med..

[76] Mellor J., Christie R., Overton C.E., Paton R.S., Leslie R., Tang M., Ward T. (2023). Forecasting Influenza Hospital Admissions within English Sub-Regions Using Hierarchical Generalised Additive Models. Commun. Med..

[77] World Health Organization (2024). Influenza Public Health Research Agenda, Update 2024.

[78] World Health Organization (2024). Maintaining Essential Health Services: Operational Guidance for the COVID-19 Context.

